# Nanosecond Pulsed Electric Field Induces an Antitumor Effect in Triple-Negative Breast Cancer via CXCL9 Axis Dependence in Mice

**DOI:** 10.3390/cancers15072076

**Published:** 2023-03-30

**Authors:** Zhentian Xu, Caixu Pan, Luyan Chen, Junjie Qian, Xinhua Chen, Lin Zhou, Shusen Zheng

**Affiliations:** 1 Division of Hepatobiliary and Pancreatic Surgery, Department of Surgery, The First Affiliated Hospital, Zhejiang University School of Medicine, Hangzhou 310003, China; 2 NHC Key Laboratory of Combined Multi-Organ Transplantation, Hangzhou 310003, China; 3 Key Laboratory of The Diagnosis and Treatment of Organ Transplantation, Research Unit of Collaborative Diagnosis and Treatment for Hepatobiliary and Pancreatic Cancer, Chinese Academy of Medical Sciences (2019RU019), Hangzhou 310003, China; 4 Key Laboratory of Organ Transplantation, Research Center for Diagnosis and Treatment of Hepatobiliary Diseases, Hangzhou 310003, China; 5 Breast Surgery, The First Affiliated Hospital, Zhejiang University School of Medicine, Hangzhou 310003, China; 6 Key Laboratory of Pulsed Power Translational Medicine of Zhejiang Province, Hangzhou 311121, China

**Keywords:** nanosecond pulsed electric field, apoptosis, immune response, CXCL9 axis, triple-negative breast cancer

## Abstract

**Simple Summary:**

Triple-negative breast cancer is an aggressive malignancy with a poor prognosis. Nanosecond pulsed electric field is a new method for local tumor ablation. However, the mechanism of nanosecond pulsed electric field therapy for triple-negative breast cancer is not clear. We constructed a mouse model of triple-negative breast cancer. Transcriptome sequencing and other techniques were used. We demonstrated that nanosecond pulsed electric field effectively ablated tumor and induced apoptosis related pathways. Nanosecond pulsed electric field ablation activated tumor immunity and promoted the infiltration of CD8^+^ T cells. The inhibition of residual cancer growth mediated by nanosecond pulsed electric field ablation was dependent on CXCL9 axis.

**Abstract:**

Triple-negative breast cancer (TNBC) is a refractory tumor, and therapeutic options are very limited. Local ablation has been applied recently. Chemokines play a critical role in the recruitment of immune cells into ablative tumors. Nanosecond pulsed electric field (nsPEF) shows potential anti-tumor efficacy, but the mechanism for maintaining the immune effect is not very clear. Here, we applied nsPEF for treating 4T1 breast cancer cells in vitro. RNA sequencing (RNA-seq) was applied. Anti-CXCL9 was used alone or combined with nsPEF to treat triple-negative breast cancer in mice. We demonstrated that nsPEF effectively induced cell apoptosis and inhibited the growth and metastasis of triple-negative breast cancer. An immune effect, especially chemotaxis, was activated by nsPEF. The number of infiltrated CD8^+^ T cells was increased significantly. We found that the inhibition of residual breast cancer growth by nsPEF was dependent on the CXCL9 axis. In conclusion, our work demonstrated that nsPEF effectively ablated the tumor, aroused an immune response, and inhibited residual breast cancer growth via CXCL9 axis dependence in mice.

## 1. Introduction

Triple-negative breast cancer (TNBC) is characterized by aggressiveness and poor prognosis, and it has long been a refractory disease for which therapeutic choices are limited [1]. Anthracycline- and taxane-based chemotherapy are the standard treatments for females with early-stage and advanced-stage TNBC [2,3]. Nevertheless, the 5-year survival rate is less than 30% of females with metastatic breast cancer [4]. Local ablation methods, including radiofrequency ablation (RFA), microwave ablation (MWA), and cryoablation (CA), are gradually being valued. The achievement of complete ablation rates and diverse immune effects has been reported [5]. Local treatment has shown a promising prospect.

Nanosecond pulsed electric field (nsPEF), a new condensed energy originating from aerospace and military technology, can ablate tumors via the electroporation effect [6]. Through ultra-short pulses (nanosecond duration), nsPEF has the ability to penetrate cell membranes and act on organelles, which causes cell apoptosis and necrosis events, such as the release of cytochrome c, the activation of calcium ion and caspase, the generation of reactive oxygen species, and DNA breakage [7,8,9]. Apoptotic cells expose or release molecules on the surface as ‘find me’ signals to attract phagocytes [10]. Chemokines are critical for the recruitment of immune cells [11]. Chemokines can be divided into four types according to their structure: (1) CC–chemokines, (2) CXC–chemokines, (3) C–chemokines, and (4) CX3C–chemokines [12]. CXCL9 facilitates the infiltration of effector T cells and NK cells into the tumor [12]. NsPEF was found to induce antitumor immunity in breast cancer [13]. However, the mechanism underlying nsPEF’s maintenance of the immune effect remains unclear.

According to the principle of irreversible electroporation, we independently developed a nanosecond pulsed tumor ablation system supported by the National S&T Major Project of China (2018ZX10301201). In this study, we investigated the efficacy and apoptosis induction of nsPEF in triple-negative breast cancer in mice. We further explored the immune mechanism of nsPEF inhibiting residual breast tumor growth. We found that nsPEF could induce strong apoptosis of triple-negative breast cancer in mice and an effective immune response. We also demonstrated that the inhibition of residual breast cancer growth by nsPEF was dependent on the CXCL9 axis.

## 2. Materials and Methods

### 2.1. Cell Culture

The murine mammary carcinoma 4T1 cell line was purchased from the Chinese Academy of Sciences (Shanghai, China). The cells were well-cultured with RPMI-1640 medium (Biological Industries, Kibbutz Beit HaEmek, Israel) containing 10% fetal bovine serum (GIMINI, Woodland, CA, USA) in a 37 °C, 5% CO_2_ incubator. The medium was changed once per day. Cells were passaged when the cell density exceeded 85%.

### 2.2. Detection of Apoptosis by Flow Cytometry

The 4T1 cells in the logarithmic growth stage were digested by trypsin and prepared into single-cell suspensions with appropriate concentrations. Cell suspensions were added into different shock cups treated with different field strengths (50 pulses with 0, 20, or 30 kV/cm). The treated cell suspensions were divided into 0 h and 24 h groups. The 200 μL cell suspensions in each cup were then transferred to new flow tubes. Three repetitions for each parameter processing group were set. The annexin V/FITC apoptosis kit (Dojindo Laboratories, Kumamoto, Japan), with 5 μL of annexin V/FITC-binding solution and 5 μL of PI solution, was implemented. The cells were gently shaken and mixed with 100 μL of 1× annexin V-binding solution. Apoptosis was detected by flow cytometry (BD, San Jose, CA, USA).

### 2.3. Inoculation of Tumor and Tumor Ablation by nsPEF

**Figure 1:**
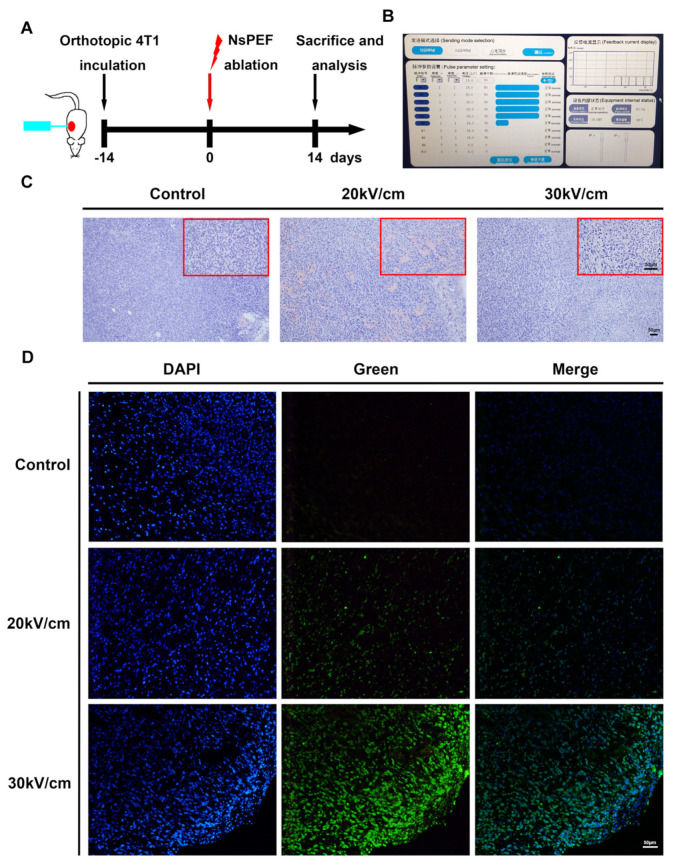
NsPEF induces apoptosis of solid tumors. (**A**) Flow chart of animal ablation. (**B**) Working interface of the nanosecond pulsed tumor ablation system. (**C**) HE staining of tumors in the control group, 20 kV/cm group, and 30 kV/cm group at 0 h after nsPEF treatment. The larger HE images were captured under a 10× objective, while the smaller images were captured under a 40× objective. Scale bars: 50 μm. (**D**) TUNEL essay at 0 h by nsPEF (magnification: 20× objective; scale bar: 50 μm).

Female Balb/c mice (5–6 weeks old) were purchased from SLAC (Shanghai SLAC Laboratory Animal Co., Ltd., Shanghai, China) and kept in an SPF animal room in the Animal Center, the First Affiliated Hospital, School of Medicine, Zhejiang University. All the experiments were approved by the Ethics Committee for Animal Experimentation at the School of Medicine, Zhejiang University and were strictly in accordance with the National Institutes of Health Guide. The concentration of 4T1 cell suspensions was 5 × 10^6^ cells/mL. Firstly, 5 × 10^5^ cells were inoculated into the right–second mammary gland of the mouse. After 2 weeks, the subcutaneous tumors were measured by vernier caliper to be about 0.8 cm. Mice were divided into the control group (0 kV/cm), 20 kV/cm group, and 30 kV/cm group (*n* = 12). The nanosecond pulsed tumor ablation system was applied (Hangzhou Ruidi Biotechnology Co., Ltd., Hangzhou, China) (Figure 1B). In the nsPEF ablative groups, the parameters were as follows: duration, 300 ns; frequency, 2 Hz; distance between needles, 0.5 cm; 400 pulses (two passes with vertical intersecting placement of needles, 200 pulses each) at 20 or 30 kV/cm field strength (Figure S1A). The growth states of mice and tumors were regularly observed. According to the animal ethics and welfare regulations, animals were euthanized in cases of the tumor breaking or growing to >2.0 cm. Mice were euthanized 14 days later (Figure 1A). The tumor volume (V) was calculated according to the formula V (mm^3^) = ab^2^/2, where a is the maximum length and b is the minimum length.

### 2.4. Experiments of CXCL9 Deletion in Animals

Mice with 5 × 10^6^ 4T1cells/mL inoculated in situ were randomly divided into the control group, anti-CXCL9 group, nsPEF group and anti-CXCL9 + nsPEF group on day 14. We then administered 250 μg of anti-mouse CXCL9 (BE0309, BioXcell, West Lebanon, NH, USA) twice per week to each mouse in the Anti-CXCL9 and Anti-CXCL9 + nsPEF groups. The nsPEF and anti-CXCL9 + nsPEF groups were ablated with 50 pulses for 300 ns at 2 Hz. All mice were observed regularly and euthanized on the 18th day after first treatment.

### 2.5. Hematoxylin and Eosin (HE) Staining and Immunohistochemistry (IHC)

The mice were euthanized, and the tumors at 0 h and tissues at day 14 after ablation were fixed in 4% paraformaldehyde. All tissues were embedded in paraffin and then sliced into sections of 3–4 μm. Hematoxylin and eosin (HE) staining, including hydration, dying, dehydration, and sealing, was applied. CD8 (98941, CST, Danvers, MA, USA) and Granzyme B (44153, CST, USA) antibody were purchased for IHC. Slices were observed under a microscope (Olympus, Tokyo, Japan).

### 2.6. TUNEL

Cleaved DNA exposed to 3’-OH can be bound to FITC-labeled dUTP catalyzed by terminal deoxynucleotidyl transferase (TdT). Paraffin sections of the tumor at 0 h were prepared to detect the apoptosis using a one-step TUNEL apoptosis detection kit (C1088, Beyotime, Shanghai, China) according to the instructions. The slices were sealed and observed using an inverted fluorescence microscope (OLYMPUS IX81, Tokyo, Japan).

### 2.7. Transcriptome Analysis and Infiltrated Immune Cells Analysis

Two weeks after inoculation of 4T1 cells, the mice were divided into the control and nsPEF groups. The nsPEF parameters were as follows: 300 ns, 2 Hz, 50 pulses, and 30 kV/cm. After 7 days, the RNA sequencing (RNA-seq) analysis was carried out in collaboration with Gene Denovo Biotechnology Co., Ltd. (Guangzhou, China). The screening conditions for significantly different genes were as follows: *p* values < 0.05 and FC > 1.2. Bioinformatic analysis was accomplished using Omicsmart—an online platform for data analysis (https://www.omicsmart.com (accessed on 1 November 2022)). The CIBERSORTx online tool was used for statistical analysis (https://cibersortx.stanford.edu (accessed on 17 February 2023)) based on the RNA-seq data.

### 2.8. Inflammatory Cytokine Micro-Array

Three days after ablation as described in Section 2.6, the tumors of mice in the control and nsPEF groups were harvested and stored in a −80 °C refrigerator for the Quantibody Mouse Inflammation Array 1 (QAM-INF-1, RayBiotech, Norcross, GA, USA) test.

### 2.9. Flow Cytometry

Antibodies corresponding to Biolegend were purchased for Zombie NIR Fixable Viability Kit (423106), CD45 (103112, 103140), CD3 (100218), CD4 (100510), CD8 (100706), CD11b (101245), F4/80 (123108) and CXCL9 (515606). The tumor was digested and ground. White blood cells were isolated using OptiPrep density gradient medium (D1556, Sigma-Aldrich, St. Louis, MO, USA). The Zombie NIR Fixable Viability Kit was incubated at room temperature for 15 min and then with the corresponding surface antibodies at 4 °C for 20 min, before resuspending in PBS. Isolated leukocytes were resuspended and counted with 10% FBS 1640 medium containing Leukocyte Activation Cocktail (550583, BD, USA); they were then cultured at 37 °C in the 96-well plate for 6 h. After incubation with the Zombie NIR Fixable Viability Kit and surface antibodies, cells were fixed using a Fixation/Permeabilization Kit (554714, BD, USA) and incubated with CXCL9 antibodies. Cells were tested by flow cytometry (BD FACSCantoTM II, BD, USA) or (Fortessa, BD, USA). The results were analyzed using FlowJo 10.8 software.

### 2.10. Detection of CXCL9 by ELISA

CXCL9 in the tumor was detected using a Mouse CXCL9/MIG ELISA Kit (KE10067, Proteintech, Chicago, IL, USA) according to the instructions.

### 2.11. Statistical Analysis

Data were analyzed using GraphPad Prism 8 (GraphPad Software, La Jolla, CA, USA) and are presented as the mean ± SEM. The *p*-values were assessed using Student’s *t*-tests, as well as one-way and two-way ANOVA analysis. A *p*-value of < 0.05 was considered significant.

## 3. Results

### 3.1. NsPEF Induces Apoptosis of Solid Tumor

According to the endurance capacity of mice and the electric field parameters of previous studies [7,14,15,16], we set 0 kV/cm (control group), 20 kV/cm, or 30 kV/cm for ablation (Figure 1B). Following the application of 400 pulses for 300 ns at 2 Hz, HE staining at 0 h suggested that, unlike the tumors in the control group with large numbers of mitotic cells, nsPEF induced tumor cell death. Nucleolysis and chromatin fragmentation were observed in the ablative area; both observations were more obvious in the 30 kV/cm group than in the 20 kV/cm group (Figure 1C). The TUNEL test revealed cell apoptosis. With an increase in electric field intensity, cell apoptosis increased significantly (Figure 1D). 

### 3.2. NsPEF Effectively Inhibits Tumor Growth and Distant Metastasis

**Figure 2:**
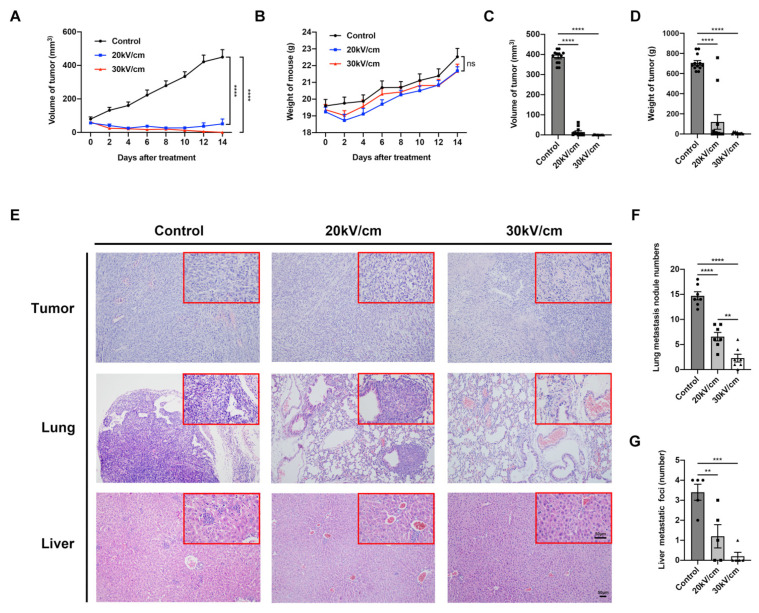
NsPEF effectively inhibits tumor growth and distant metastasis. Changes in (**A**) tumor volume (*n* = 12) and (**B**) weight of mice (*n* = 7). (**C**) Tumor volume and (**D**) tumor weight of mice in three groups 14 days after nsPEF treatment, (*n* = 12). (**E**) HE staining was performed 14 days after treatment. The larger image was captured under a 10× objective, while the smaller image was captured under a 40× objective. Both scale bars: 50 μm. (**F**) Number of lung metastasis nodules counted in mice 14 days after nsPEF, (*n* = 7). (**G**) Number of liver metastatic foci calculated under a microscope. Five tumors were taken from each group, while five fields were randomly selected for each section of tumor to count the metastatic foci under 40× magnification; the average number of foci in each section weas calculated (*n* = 5). Data are presented as the mean ± SEM; ** *p* < 0.01, *** *p* < 0.001, and **** *p* < 0.0001; ns: no significance according to one-way ANOVA.

The efficacy of nsPEF in breast cancer ablation was investigated. Tumor growth was significantly inhibited by nsPEF treatment within 14 days (*p* < 0.0001), and no significant weight loss was observed in mice (Figure 2A,B). On the 14th day, the mean tumor volume of the control group was 387.96 ± 9.52 mm^3^, whereas that of the 20 kV/cm and 30 kV/cm groups was 14.17 ± 5.87 mm^3^ and 0.94 ± 0.36 mm^3^, respectively. The tumor weight in each group was 706.08 ± 22.79 mg, 119.55 ± 72.64 mg, and 8.62 ± 2.76 mg, respectively. The tumor volumes and weights in the nsPEF groups were significantly smaller than in the control group (*p* < 0.0001 and *p* < 0.0001, respectively) (Figure 2C,D). Compared with the 20 kV/cm group, the tumor volume was smaller in the 30 kV/cm group. NsPEF could completely ablate breast tumor to a certain extent, especially in the 30 kV/cm group (Figure S1B). Furthermore, the number of lung and liver metastases in the control group was 14.71 ± 0.7 and 3.4 ± 0.4, respectively; the numbers of lung metastases in the nsPEF groups were 6.57 ± 0.73 and 2.29 ± 0.67, respectively, which were significantly reduced compared with the control group (*p* < 0.0001 and *p* < 0.0001, respectively). The number of liver metastases in the nsPEF groups was 1.2 ± 0.58 (*p* < 0.01) and 0.2 ± 0.2 (*p* < 0.001), respectively. Compared with the 20 kV/cm group, 30 kV/cm inhibited distant lung metastasis more effectively (*p* < 0.01) (Figure 2E–G and Figure S1D). These data confirmed that nsPEF, particularly at 30 kV/cm, could effectively ablate 4T1 breast tumors and reduce distant metastasis, as well as achieve complete tumor ablation to a certain extent.

### 3.3. Fifty Pulses of nsPEF Are Sufficient to Induce High Level of Apoptosis in 4T1 Cells

**Figure 3:**
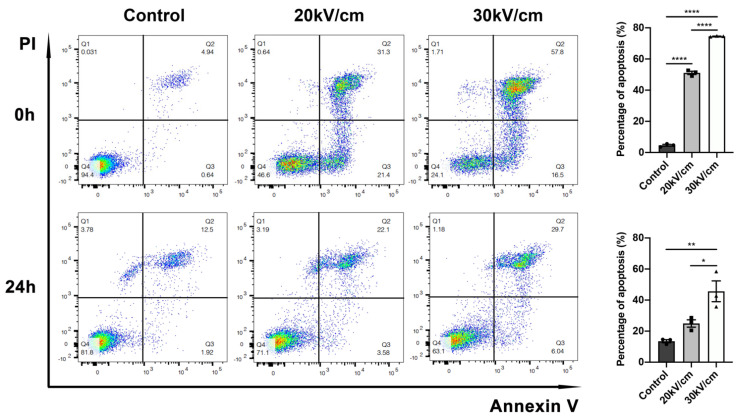
Fifty pulses of nsPEF were sufficient to induce a high level of apoptosis in 4T1 cells. The apoptosis of 4T1 cells in vitro was detected by flow cytometry at 0 h and 24 h. The total apoptotic rates at two timepoints are shown on the right (*n* = 3). Data are presented as the mean ± SEM; * *p* < 0.05, ** *p* < 0.01, and **** *p* < 0.0001; ns: no significance according to one-way ANOVA.

We verified the ability of nsPEF to ablate tumors, and we selected 30 kV/cm as a safe and appropriate parameter in the above experiments. Because tumors are irregular, nanosecond pulsed electric fields or local ablation methods such as radiofrequency ablation can more or less result in residual cancer. To better simulate incomplete ablation, we reduced the number of nanosecond pulses to 50. We then investigated whether a low pulse count of 50 caused apoptosis. Compared with the control group, early and late apoptosis distinctly occurred in the nsPEF group. The total apoptosis rates in the 20 kV/cm and 30 kV/cm groups were significantly increased at 0 h (*p* < 0.0001 and *p* < 0.0001, respectively), with the effect of 30 kV/cm being more significant than that of 20 kV/cm (*p* < 0.0001). Furthermore, 30 kV/cm retained the effect of inducing apoptosis at 24 h (30 kV/cm vs. control, *p* < 0.01); (30 kV/cm vs. 20 kV/cm, *p* < 0.05); (Figure 3).

### 3.4. NsPEF Activates Apoptosis-Related Pathways

**Figure 4:**
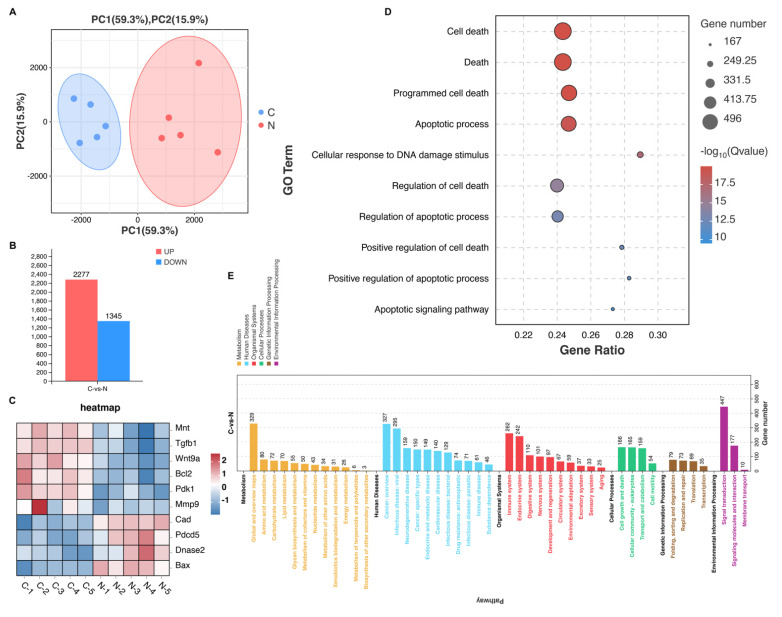
NsPEF promotes the activation of apoptosis-related pathways. (**A**) Principal component analysis (PCA) of the control group (**C**) and nsPEF group (N). (**B**) Statistical map of differential genes. (**C**) Differential expression heatmap of apoptosis-related genes. (**D**) Gene Ontology (GO) analysis associated with cell death. (**E**) Kyoto Encyclopedia of Genes and Genomes (KEGG) enrichment analysis.

To testify whether fifty pulses of nsPEF at 30 kV/cm induced the activation of apoptosis-related pathways during solid tumor ablation, we removed the tumors in the control group and nsPEF group on day 7 for transcriptome sequencing. Principal component analysis (PCA) analysis showed good homogeneity of samples in the two groups (Figure 4A). Compared with the control group, 2277 genes were upregulated and 1345 genes were downregulated (Figure 4B). Genes that promoted growth or inhibited apoptosis such as Mnt, Tgfb1, Wnt9a, Bcl2, Pdk1 and Mmp9 were significantly downregulated in the nsPEF group. In contrast, genes associated with the process of apoptosis, such as Cad, PDCD5, Dnase2, and Bax, were significantly upregulated in the nsPEF group (Figure 4C). Gene ontology (GO) analysis showed that the death-related pathways, especially the apoptotic pathway, were obviously enriched (Figure 4D). NsPEF induced strong biological effects, such as metabolism and signal transduction (Figure 4E), as well as modulating the immune system (Figure 4E). 

### 3.5. NsPEF Induced the Release of Inflammatory Cytokines and the Infiltration of Immune Cells into Tumor

**Figure 5:**
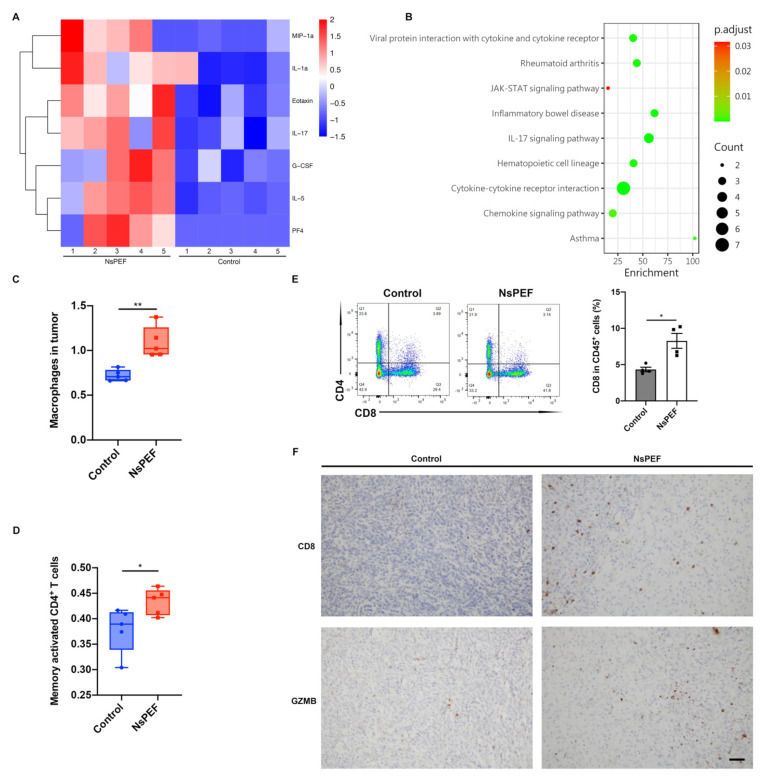
NsPEF activated inflammatory pathways and promoted the activation of immune cells. (**A**) Inflammatory factor micro-array of tumors in the nsPEF group and control group on the third day after treatment; heatmap showing the changes in cytokines (*n* = 5, *p* < 0.05). (**B**) KEGG pathway enrichment analysis of cytokines. (**C**) Macrophages and (**D**) memory activated CD4^+^ T cells in tumor on day 7 according to the results of CIBERSORTx based on the RNA-seq data. (**E**) Changes in the proportion of CD8^+^ T cells in CD45^+^ cells in the control group and the nsPEF group (*n* = 4). The flow plot showed the proportion of CD8^+^ T cells in the CD3^+^ T cell gate. (**F**) Immuno-histochemistry (IHC) of CD8 and GZMB (Granzyme B) in tumors of the two groups on day 7. Scale bar: 50 μm. Data are presented as the mean ± SEM; * *p* < 0.05, ** *p* < 0.01, according to Student’s *t*-test.

When tumor cells go through apoptosis, they release a “find me” signal to recruit phagocytes for phagocytosis, thus triggering a cascade of immune responses [10]. We sought to determine whether our nanosecond pulsed tumor ablation system could also trigger an immune response. Firstly, we tested the cytokines of tumors in the control and nsPEF groups on day 3 after ablation. The inflammatory cytokine microarray showed that G-CSF, IL-1A, IL-17, and other cytokines in the tumor in the nsPEF group were significantly increased on the third day (*p* < 0.05) (Figure 5A), while inflammatory pathways were activated (Figure 5B); these results indicated chemotaxis and infiltration of inflammatory cells into the tumor. On the basis of RNA-seq data, we used the CIBERCORTx online tool to analyze the immune cells in the tumor. We found that macrophages and memory-activated CD4^+^ T cells were significantly elevated in tumor from the nsPEF group (*p* < 0.01 and *p* < 0.05, respectively) (Figure 5C,D). Infiltrated CD8^+^ T cells were significantly increased in the nsPEF group (*p* < 0.05) (Figure 5E). Furthermore, IHC indicated that CD8^+^ T cells achieved greater infiltration into the tumor after nsPEF ablation, whereas they were mainly distributed along the margin of the tumor in the control group. NsPEF induced a greater number of cytotoxic CD8^+^ T cells producing Granzyme B in the tumor (Figure 5F).

### 3.6. NsPEF Inhibits the Growth of Residual Breast Cancer via CXCL9 Axis Dependence

**Figure 6:**
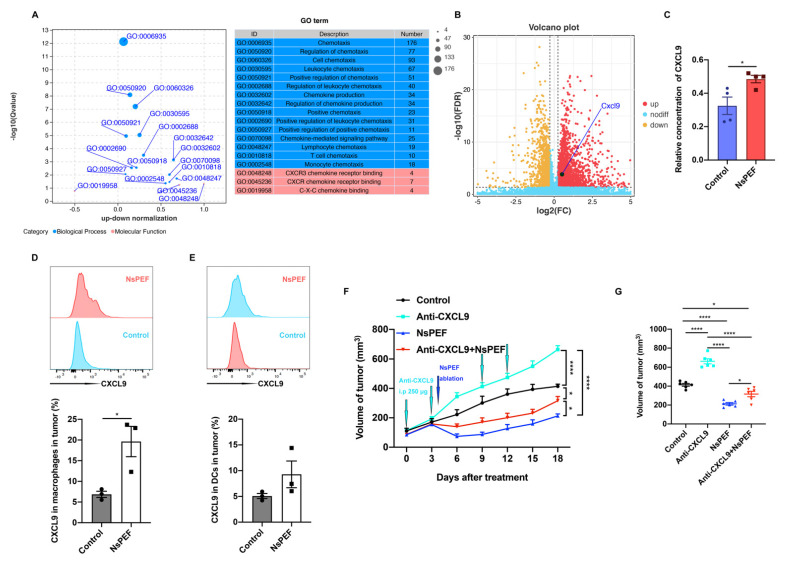
NsPEF inhibited the growth of residual breast cancer via CXCL9 axis dependence in mice. (**A**) Bubble diagram of GO enrichment. (**B**) Volcano plot of differential genes. (**C**) Relative concentration of CXCL9 in tumor tissues belonging to the control and nsPEF groups according to ELISA. Expression of CXCL9 in (**D**) macrophages and (**E**) DCs (dendritic cells) in tumor (*n* = 3). (**F**) Changes in tumor volume of mice in the control, anti-CXCL9, nsPEF, and anti-CXCL9 + nsPEF groups (*n* = 6). (**G**) Tumor volumes on day 18 after treatment in the control, anti-CXCL9, nsPEF, and anti-CXCL9 + nsPEF groups (*n* = 6). Data are presented as the mean ± SEM; * *p* < 0.05, **** *p* < 0.0001; ns: no significance according to Student’s *t*-test or one-way ANOVA.

Chemokines are essential parameters for recruiting immune cells. We found that nsPEF induced the immune effects through KEGG analysis (Figure 4E). Moreover, chemotaxis was highly induced by nsPEF according to GO analysis (Figure 6A). Elevated levels of CXCL9 correlate with increased tumor infiltration of CD8^+^ T cells and tumor suppression [12]. CXCL9 was more significantly expressed in the nsPEF group (*p* = 0.00016) (Figure 6B). We found that the relative concentration of CXCL9 in tumor tissues was significantly increased after nsPEF treatment (*p* < 0.05) (Figure 6C). Macrophages in the nsPEF group expressed more CXCL9 than those in the control group (*p* < 0.05) (Figure 6D). Dendritic cells (DCs) in the nsPEF group also relatively increased the expression of CXCL9 (Figure 6E). We then applied for the anti-CXCL9 antibody in vivo to verify the role played by CXCL9. Tumors in the anti-CXCL9 group increased in size more quickly than those in the control group (*p* < 0.0001) (Figure 6F,G). NsPEF inhibited the growth of residual tumors significantly compared with the anti-CXCL9 group and the control group (*p* < 0.0001 and *p* < 0.0001, respectively). When combined with the anti-CXCL9 antibody, the efficacy of nsPEF in suppressing residual tumor growth was reduced. The above results demonstrated that nsPEF’s inhibition of residual tumor growth was dependent on the CXCL9 axis.

## 4. Discussion

NsPEF has shown promising potential in many tumor treatments due to its non-thermal advantage and ablative ability, which are hardly affected by the “heat-sink” effect, unlike traditional thermal ablation [17,18,19,20]. In many cancers, pro-apoptotic proteins are mutated and inactivated, or anti-apoptotic proteins are upregulated, resulting in uninhibited tumor growth [21]. When cells are stressed or damaged, the apoptosis process is initiated [22]. Bcl2 family members located in the mitochondria can inhibit or stimulate the release of cytochrome c, thereby inhibiting or promoting cell apoptosis [23]. Overexpression of Bcl2 inhibits the process of apoptosis, while Bax and Bak promote cell death [23,24]. Protein damage causes the aggregation of Bax and Bak, resulting in the release of pro-apoptotic factors, such as Smac and cytochrome c in the mitochondria [25]. We found that the expression of genes related to apoptosis and DNA degradation, such as Cad, PDCD5, Dnase2 and Bax, increased after nsPEF ablation of triple-negative breast cancer in mice. NsPEF induced significant apoptosis. When cells undergo apoptosis, some molecules are released as a signal to initiate immunity. Several studies have reported that nsPEF resulted in the release of damage-associated molecular patterns (DAMPs), such as ATP, calreticulin (CRT), and high mobility group box 1 (HMGB1), to promote immune responses [26,27]. RNA-seq showed that nsPEF caused immune process, especially the chemokine-related pathways. Chemokines recruit immune cells to enter the tumor for an anti-tumor response. CXCL9 induced by IFN-γ primarily mediates lymphocyte infiltration into lesions and inhibits tumor growth [28]. The CXCL9, -10, -11/CXCR3 axis chiefly regulates the migration, differentiation, and activation of immune cells [29]. CXCL9 and CXCL10 also have the function of inhibiting endogenous tumor angiogenesis [30]. After nsPEF treatment, the level of CXCL9 in the tumor was significantly upregulated, and the secretion of CXCL9 by antigen-presenting cells, such as macrophages and DCs, was increased. The number of cytotoxic CD8^+^ T cells also increased. Studies have shown that incomplete ablation of radiofrequency ablation can promote tumor progression, which is not conducive to long-term survival of patients [31]. We found that nsPEF effectively ablated triple-negative breast cancer in mice and inhibited the growth of residual cancer. The mechanism of inhibition of residual cancer was dependent on the CXCL9 axis. The limitation of this study is that we did not deeply explore the role that CXCL10 and regulatory cells play, which will be addressed in the future.

## 5. Conclusions

We verified that our independently developed ablation system has a good therapeutic effect. NsPEF could effectively induce the apoptosis of mammary triple-negative breast cancer cells, as well as inhibit their growth and distant metastasis. NsPEF promotes chemokine-related pathways and an immune response. For the first time, we found that nsPEF’s growth inhibition of residual triple negative breast cancer cells in mice was dependent on CXCL9 axis. This provides a new perspective for local treatment combined with immunotherapy or chemotherapy.

## Data Availability

The RNA-seq data are not publicly available due to some of the data needs to be further used.

## Supplementary Materials

The following supporting information can be downloaded at: https://www.mdpi.com/article/10.3390/cancers15072076/s1, Figure S1. NsPEF ablated solid tumors. (A) Photo of nsPEF ablating mouse tumors. (B) Changes in tumor of mice 14 days after ablation. (C) Mouse tumors extracted 14 days after ablation. (D) Lung metastasis nodules counted 14 days after ablation. The ones in the green circle was the metastatic nodule.
