# Supplementary material for: Nanosecond Pulsed Electric Field Induces an Antitumor Effect in Triple-Negative Breast Cancer via CXCL9 Axis Dependence in Mice
Source: Cancers (Basel). 2023 Mar 30;15(7):2076. doi: 10.3390/cancers15072076 (PMC10093317; doi:10.3390/cancers15072076)
Supplement: Supplementary file 1 [file cancers-15-02076-s001.zip › cancers-2273488-supplementary.pdf]

cancers

MDPI

Nanosecond Pulsed Electric Field Induces an Antitumor Effect in Triple-Negative Breast Cancer via CXCL9 Axis Dependence in Mice

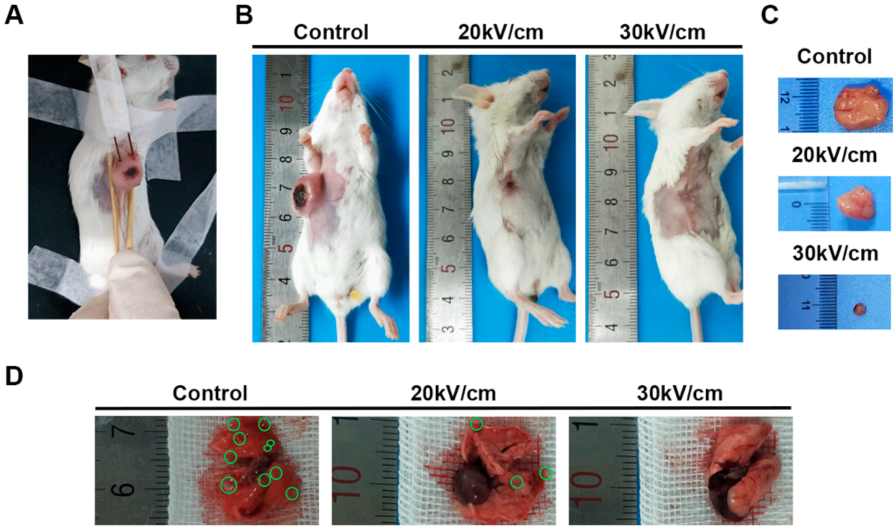

Figure S1. NsPEF ablated solid tumors. (A) Photo of nsPEF ablating mouse tumors. (B) Changes in tumor of mice 14 days after ablation. (C) Mouse tumors extracted 14 days after ablation. (D) Lung metastasis nodules counted 14 days after ablation. The ones in the green circle was the metastatic nodule.
